# Analysis of environmental factors influencing lumpy skin disease outbreak seasonality and assessment of its spread risk in the Saratovskaya oblast of Russia

**DOI:** 10.14202/vetworld.2024.630-644

**Published:** 2024-03-21

**Authors:** Dmitry Podshibyakin, Larisa Padilo, Valery Agoltsov, Oleg Chernykh, Olga Popova, Kalabekov Mutalif, Nataliya Solotova

**Affiliations:** 1 Scientific Research Institute of Organic and Inorganic Chemistry Technologies and Biotechnology LLC, Saratov, Russia; 2 Department of Veterinary Medicine and Biotechnology, Saratov State University of Genetics, Biotechnology and Engineering Named after N.I. Vavilov, Saratov, Russia; 3 Department of Microbiology and Animal Virology, Kuban State Agrarian University Named after I.T. Trubulin, Krasnodar, Russia; 4 Department of Animal Management and Veterinary-Sanitary Expertise, Kabardino-Balkaria State Agrarian University Named after V.M. Kokov, Nalchik, Russia

**Keywords:** cattle, environmental factors, generalized linear regression, lumpy skin disease, maximum entropy, species distribution

## Abstract

**Background and Aim:**

Lumpy skin disease (LSD) is a transboundary viral disease of cattle that causes serious economic losses due to a significant decrease in meat and milk productivity. This study analyzed the influence of natural and anthropogenic environmental factors on LSD spread seasonality and assessed the risk of LSD outbreaks in the Saratovskaya oblast of the Russian Federation.

**Materials and Methods:**

Data on LSD outbreaks and environmental factors during different seasons were collected for the period 2011–2020 in the Balkan Peninsula, Middle East, and Russia. Risk assessment was performed using mathematical modeling with generalized linear regression and maximum entropy.

**Results:**

Fourteen statistically significant environmental factors influencing LSD spread were identified. The analysis of MaxEnt models built using the selected factors showed that the presence of the pathogen is mostly exerted by: the density of susceptible cattle (an increased risk is observed at a density above 10 and 20 heads/10 km^2^ in winter and autumn, with a permanent risk in spring and summer), the density of water bodies (the risk is increased at any density in winter and autumn, in the range of 13–23.5 m^2^/km^2^ in spring, in the ranges of 0–8 and over 14.5 m^2^/km^2^ in summer), and average monthly precipitation rate (the most risky are 105–185 mm/month in winter, 35 mm in spring, 15–105 mm in summer, and above 50 mm in autumn).

**Conclusion:**

LSD tends to spread during the warm season. Compared with other test zones, the Saratovskaya oblast has a negligible risk of disease spread (in winter), low risk (in spring), or medium risk (in summer and autumn). The annual risk is low to medium.

## Introduction

Lumpy skin disease (LSD) is a transboundary viral disease of cattle (bovine) characterized by lesions of the conjunctiva, genital organs, and mucous membranes of the respiratory and digestive tracts. LSD is accompanied by fever and the formation of skin nodules with subsequent necrosis. The causative agent of the disease is a DNA-containing *Capripoxvirus* of the family *Poxviridae* [1–4]. Despite the relatively low mortality, this disease causes serious economic losses due to a significant decrease in the meat and milk productivity of cattle, the quality of raw hides, the development of sterility of sires and abortions of cows, the cost of vaccination, and the death of sick animals caused by a secondary infection [1, 4, 5].

The significance of the study was determined by the steady and rapid expansion of the LSD nosoarea. It was originally endemic to Africa, and a large number of outbreaks were registered in the Middle East, the Balkans, Central and Eastern Asia, and the Commonwealth of Independent States. To date, it has been established that the spread of this disease is mainly associated with the transport of infected animals and the activity of blood-sucking *Arthropoda* species in livestock farms. These factors determine the longevity of the disease agent within the nosoarea and its introduction into new territories, creating the risk of pathogen rooting and future outbreaks of the disease [3, 6–8].

This study aimed to analyze the influence of various environmental factors on the seasonality of LSD spread using retrospective data on reported outbreaks of the disease in the most affected regions of Europe, the Middle East, and Russia between 2011 and 2020 and to assess the risk of spread of this disease in the Saratovskaya oblast (Russia).

Unlike other studies [4, 8] that examined factors influencing LSD spread, this study used a longer period of time, which contributed to more precise statistics and mathematical modeling using the methods of generalized linear regression and maximum entropy, which allowed us to determine three factors having the utmost impact on the process of LSD spread in five test zones, specifically in the Saratovskaya oblast of Russia.

## Materials and Methods

### Ethical approval

This study did not require ethical approval because no animal or human subjects were involved.

### Study period and location

The study was conducted from July 2022 to April 2023 at the Saratov State University of Genetics, Biotechnology, and Engineering, named after N.I. Vavilov (Saratov, Russia).

### Datasets

It is extremely important to choose environmental factors of the test areas to model changes in the nosoarea of pathogens of transmissive diseases and assess the risk of their spread.

The selected factors should be relevant in the time the outbreak was recorded, correspond to the spatial scale of the research, and be ecologically relevant to the pathogen, its vectors, and hosts [9–11].

The factors suggested in this research can be divided into three categories: natural, anthropogenic, and land cover, defined as the physical material at the surface of the Earth, formed jointly by both natural and anthropogenic (land use) influences.

The first category includes elevation above sea level, climatic factors (temperature, precipitation rate, average wind speed in a given area, etc.), level of solar radiation, and the presence of open water (distance to the nearest water body and the density of water bodies in the territory). These factors are characterized by significant seasonal changes; therefore, LSD outbreak risk assessment was carried out separately for the year as a whole and for each season.

Two datasets were used as sources of information on climatic factors. The first set of the Climatic Research Unit gridded Time Series (CRU TS) 4.05 dataset (https://crudata.uea.ac.uk/cru/data/hrg/cru_ts_4.05/, accessed: December 22, 2023) [12] includes a series of average climatic variables for each month of each year on the territory of all continents except Antarctica with a resolution of 0.5 arc degrees (≈ 25 km^2^ at the equator). On the basis of this information, the average annual and quarterly climatic indicators of 2011–2020 were calculated and then used in the analysis. The second set, retrieved from the WorldClim v.2.1 weather and climate database by Stephen E. Fick and Robert J. Hijmans, University of California, USA (https://www.worldclim.org, accessed: December 22, 2023), contains nineteen bioclimatic variables with a resolution of 30 arc seconds (≈ 1 km^2^ at the equator) for all continents based on observations from 1970 to 2000 [13]. This dataset was used to analyze the influence of climatic factors throughout the year, except for data on the average annual temperature (variable BIO1) and average annual precipitation (BIO12) since they were calculated based on information from the CRU TS 4.05 dataset.

To calculate the average annual and quarterly indicators of solar radiation and wind speed, the WorldClim v.2.1 database with a spatial resolution of 30 arcsec was used. Information about the average elevation above sea level was obtained from the same source.

The distance to the nearest water body and the density of the hydro network were calculated on the basis of the Global River Classification Framework (https://www.hydrosheds.org/products/gloric, accessed: December 22, 2023) [14]. All types of water bodies in the study zones were used to calculate both indicators, including temporary water bodies.

The second category includes factors related to human activities, such as the density of cattle and the density of road networks. The Food and Agriculture OrganizationGridded Livestock of the World 2.01 Database (https://tinyurl.com/26jh7pzj) [15] was used as a source of information on the density of cattle in the world, and the density of cattle in the Saratovskaya oblast was calculated on the basis of the official publicly available source of the Federal State Statistics Service [16]. The cattle density in the oblast was calculated as the ratio of the median number of cattle for the period 2011–2020 in a particular municipal district to the area of that district. Information on road network density was obtained from the Global Roads Inventory Project 4 [17].

The third category of factors included land cover. The dominant land cover type data were taken from the Food and Agriculture Organization Global Land Cover SHARE Database (https://tinyurl.com/47f25bsu). This dataset contains information on 11 types of land cover in a spatial resolution of ≈ 1 km^2^ at the equator for the entire world, except Antarctica (Table-1) [18].

**Table-1:** Land cover types used in the education of LSD spread risk models [18].

Number in dataset	Land cover type
1	Artificial Surfaces
2	Cropland
3	Grassland
4	Tree Covered Areas
5	Shrubs Covered Areas
6	Herbaceous vegetation, aquatic or regularly flooded
7	Mangroves
8	Sparse vegetation
9	Bare soil
10	Snow and glaciers
11	Waterbodies

Information on LSD outbreaks was obtained from official public sources: The World Animal Health Information System of the World Organization for Animal Health [19], the Global Animal Disease Information System (EMPRES-i) [20], and the Federal Service for Veterinary and Phytosanitary Supervision [21]. This study used base maps from NextGIS Database (NextGIS, Russia, https://data.nextgis.com/en) [22] and GADM 4.1 Database by Robert J. Hijmans (https://gadm.org/index.html) [23].

### Statistical analysis and modeling methods

All information about factors that can influence the LSD spread risk was obtained as raster geospatial datasets. These datasets were drawn to an identical geographic extent in the WGS84 coordinate system (National Geospatial-Intelligence Agency, USA, https://tinyurl.com/9dp54f92) with a resolution of 30 arcsec (in accordance with the raster datasets’ minimum resolution) using ArcGIS Desktop 10.4 (Esri, USA) [24].

**Figure-1:**
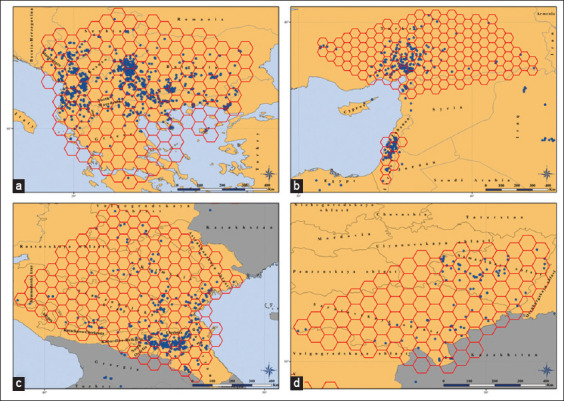
Test zones used to verify multicollinearity between the factors influencing the seasonality of LSD outbreaks, with grid cells overlaid (a-Balkan zone, b-Turkish and Israeli zones, c-South Russian zone, d-Volga zone). Blue dots are registered LSD outbreaks, and red cells are the constructed grid (Source: Base map adopted from GADM [23]).

To verify the cross-correlation (multicollinearity) between the factors considered, five test zones were identified in ArcGIS, covering the territories with the largest number of LSD outbreaks registered between 2011 and 2020. One zone has been defined on the Balkan Peninsula and encompasses all or part of Montenegro, Serbia, Kosovo, North Macedonia, Bulgaria, Albania, and Greece, as well as parts of the adjacent territory of Romania, Bosnia and Herzegovina, and Turkey. Two zones, Turkish and Israeli, have been identified in the Middle East (part of the territory of Turkey and Israel, as well as parts of the adjacent territory of Syria, Iraq, Lebanon and Jordan). Two more zones occupied part of the territory of the Russian Federation: The South Russian (covering all or most of the territory of the Krasnodarsky kray, Rostovskaya, Astrakhanskaya, Volgogradskaya oblasts, the republics of Adygea, Karachay-Cherkessia, Kabardino-Balkaria, North Ossetia, Ingushetia, Chechnya, Dagestan, Kalmykia, as well as parts of the territory of neighboring countries-Georgia and Azerbaijan) and the Volga zone (covering most of the Saratovskaya and Samarskaya oblasts, as well as parts of the territory of the adjacent subjects of the Russian Federation - the Ulyanovskaya, Penzenskaya and Orenburgskaya oblasts, and the neighboring country - Kazakhstan). The inclusion of a part of the Saratovskaya oblast in these zones made it possible to visualize the probability of the presence of an LSD pathogen in all model maps in an identical color scheme. In the ArcGIS environment, using the Repeating Shapes Tool 1.5.152 (Jenness Enterprises, USA, https://tinyurl.com/3fredjjk) [25], a grid of hexagonal cells with an area of 0.5 angular degrees (25 km^2^ at the equator) covering the territory of the outlined zones was built (Figure-1). The selected grid cell size corresponds to the highest resolution of the original raster data before geoprocessing and avoids an excessively large number of cells with no LSD outbreaks while maintaining the geospatial structure of the acting factors. The total number of cells in the constructed grid is 614. Then, using the “Zonal statistics as table” geoprocessing tool, each cell was assigned the number of LSD outbreaks, the dominant type of land cover (“Majority” computed statistic type), and the average values of other considered factors (“Mean” computed statistic type). This information is summarized in data tables (not shown) for the entire year and separately for each season. At the same time, land cover was considered a categorical factor, and all other environmental variables were considered quantitative.

Statistical processing of the obtained tabular data was performed in the R environment (https://www.r-project.org/, accessed on December 22, 2023) [26]. Because the number of reported LSD outbreaks in the cells of the grid complies with the Poisson distribution, the generalized linear models had the following form:


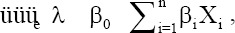


where λ is the average expected number of LSD outbreaks in a grid cell (dependent variable), log_e_(λ) is the link function that transforms the expected value of the dependent variable into a linear predictor, β_0_ is the free term, β_i_ are the regression coefficients, and X_i_ are the factor values (independent environmental variables).

The resulting models were fitted step by step. To verify the presence of outliers and influential observations of the dependent variable, leading to the overdispersion of models, the car package was used [27]. The observations were identified as outliers and influential observations if the standardized residuals from their predicted values were outside the range from –2 to 2, and Cook’s distance exceeded 1. Subsequently, they were removed from the formed datasets. Thus, five observations were removed from the winter model, 4 from the spring model, 3 from the summer model, 8 from the autumn model, and 12 from the annual model. Multicollinearity was checked in the mctest package using the variance inflation factor (VIF) test [28]. Independent variables with VIF > 10 were removed from the models.

LSD spread risk assessment based on the selected factors was performed using the maximum entropy method in MaxEnt 3.4.4 software (https://github.com/mrmaxent/Maxent) [29]. During modeling, a complementary log–log transformation was used. The results were interpreted as an index of the spatial distribution of the LSD pathogen. The value of the index (0.5) was taken as the average risk of a disease outbreak. Values above and below this index were evaluated as increased and decreased risk, respectively. The simulation was performed in 5000 iterations at a convergence threshold of 10^-5^. The maximum number of background points was 10000. A combination of linear (L), quadratic (Q), their products (P), and hinge (H) values was used as feature types. The models were tested using cross-validation in 10 replications [9, 10, 30].

To assess the quality of the constructed models, the area under the curve (AUC) of the receiver operating characteristic graph was used. AUC values can vary from 0 to 1, where 1 indicates the model’s excellent ability to distinguish the presence of the studied pathogen (i.e., outbreaks) from the background points, and a value of 0.5 corresponds to recognition at the level of randomness [31]. The quality of the models was also determined by their biological interpretability.

To assess the significance of the factors considered in predicting the probability of LSD pathogen presence, jackknife analysis and heuristic analysis included in MaxEnt 3.4.4 software (https://github.com/mrmaxent/Maxent) were used. Jackknife analysis shows the gain of AUC values for each variable when it is used separately and the lack of gain when it is excluded from the entire set of predictors. Heuristic analysis calculates the gain of each variable in the overall prediction of the spatial distribution of the virus in percentage [32, 33].

MaxEnt models were trained on the five test zones mentioned previously, where the statistical significance of environmental variables was studied. The trained models were then used to estimate the probability of the LSD pathogen spreading in these zones and the Saratovskaya oblast.

Because LSD outbreaks used to build the models are obviously confined to the range of susceptible animals and can occur repeatedly in the same locations, their spatial distribution does not withstand the test for spatial autocorrelation at the scale used in this study [11]. In this regard, we did not use Moran’s test because it is unsuitable for the data accounting method used in this study. Microsoft Excel 2016 (Microsoft Corp., Washington, USA) was used to build graphs [34].

## Results

**Figure-2:**
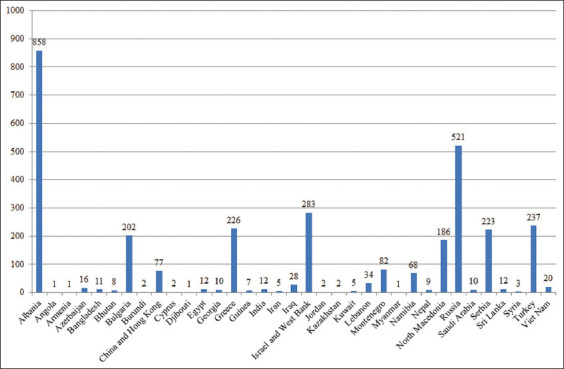
Number of LSD outbreaks registered in 2011–2020 in the territory of various states [19–21].

For the period 2011–2020, 3177 LSD outbreaks were registered worldwide (Figure-2) [19–21]. At the same time, there was a distinct seasonality of the epizootic: 254 outbreaks occurred during winter (8% of the total number), 577 outbreaks occurred in spring (18%), 1842 in summer (58%), and 504 in autumn (16%). Of the total number of outbreaks in the outlined zones, 2174 outbreaks (68.4%) occurred during the mentioned period (256 outbreaks in winter, 246 in spring, 1442 in summer, and 770 in autumn), which makes it possible to predict the risk of LSD spread to unaffected areas with a fairly high degree of accuracy using the constructed models.

Preliminary analysis of environmental factors in the test zones showed that fourteen of them were statistically significant (p < 0.001) for assessing the risk of LSD outbreaks during different seasons and throughout the year (Table-2) [12–15, 17, 18]. At the same time, five factors (the number of cattle, the road density, the dominant type of land cover, the density of water bodies, and the distance to them) passed the test for multicollinearity in the models for each season and the entire year. Three factors passed the test in four models: the diurnal temperature range and precipitation rate in all seasonal models, but not in the annual model, and the number of wet days in three seasonal models (except the summer model) and in the annual model. Two factors passed the test in the two models: the average cloud cover was statistically significant in the winter and spring models, and the average wind speed was significant in the winter and summer models. Three factors (the elevation of the area above sea level, the average number of frost days, and the average pressure of saturated water vapor in the air) were significant in only one of the seasonal models: the first factor was valid for spring, the second and third for summer. To model the LSD spread with variables calculated for the annual period, the average temperature of the wettest quarter was also a significant environmental factor.

**Table-2:** Environmental factors (independent variables) that passed the multicollinearity test in the corresponding models (p < 0.001).

Factor (independent variable)	Name in models	Model	Dimension	Reference
Cloud cover	cld_winter cld_spring	winter spring	percentage	[12]
Diurnal 2 m temperature range	dtr_winter dtr_spring dtr_summer dtr_autumn	winter spring summer autumn	°C	[12]
Frost days	frs_summer	summer	days per month	[12]
Precipitation rate	pre_winter pre_spring pre_summer pre_autumn	winter spring summer autumn	mm per month	[12]
Vapour pressure	vap_summer	summer	hPa	[12]
Wet days	wet_winter wet_spring wet_autumn wet_year	winter spring autumn annual	days per month	[12]
Elevation above sea level	Elevation	spring	meters	[13]
Mean temperature of wettest quarter	bio_8	annual	°C	[13]
Mean wind speed	Winter_mean_wind_speed Summer_mean_wind_speed	winter summer	m per second	[13]
Average distance to nearest water bodies (of all types)	Waterbodies_distance	ˍwinter spring summer autumn annual	km	[14]
Density of water bodies (of all types) network	Waterbodies_density	winter spring summer autumn annual	m^2^ per km^2^	[14]
Cattle density	Cattle_heads	winter spring summer autumn annual	total number of cattle per 10 km^2^	[15]
Density of roads (of all types) network	Roads_density	winter spring summer autumn annual	m per km^2^	[17]
Dominant type of land cover	Land_cover_dominant	winter spring summer autumn annual	–	[18]

The average values of these environmental factors in the considered zones vary within a fairly wide range. The density of cattle, for the most part, was at a minimum value of <10 heads/10 km^2^. Areas with more intensive animal husbandry (cattle density of 20–60 heads/10 km^2^) were located west of the Balkan zone, east of the Turkish zone, and south of the South Russian zone. The maximum indicator of this factor was 218 heads/km^2^ on a small stretch of the Israeli coast. The road density ranged from 0 to 25869 m/km^2^. At the same time, for most of the territory, the values of this indicator did not exceed 500 m/km^2^. Plots with higher values (up to 5000 m/km^2^) were mainly concentrated in the Balkan and Israeli zones, whereas the highest values corresponded to the location of large cities and their suburbs.

**Figure-3:**
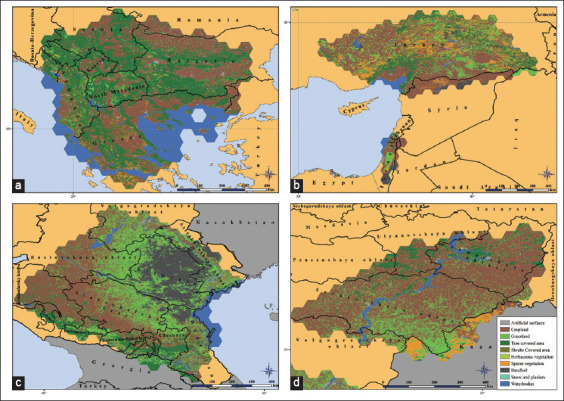
Test zones land cover: (a) Balkan zone, (b) Turkish and Israeli zones, (c) South Russian zone, and (d) Volga zone (Source: Base map adopted from GADM [23]).

The land cover of the territory of the test zones demonstrates high diversity. The largest area is occupied by cropland (41.7% of the total area), tree-covered area (18.5%), and grassland (18.4%). In addition, the test zones contain plots of bare soil (6.6%) and sparse vegetation (5.2%). Shrub-covered areas, herbaceous vegetation, and snow and glacier types of land cover are insignificant: 0.9%, 0.5%, and 0.03% of the total area of the test zones, respectively (Figure-3) [18].

The distance to water bodies (of all types) in the test zones is small and does not exceed 0.6 km. The water bodies’ network density ranges from 1.37 to 24.5 m^2^/km^2^, with the lowest level in the Israeli zone.

The elevation above sea level in the test zones varies from −420 m (Israeli zone) to 4768 m (South Russian zone). Areas with heights over 2000 m above sea level occupy only a small part of the territory and are located to the south of the South Russian and to the east of the Turkish zones. Territories with altitudes in the range of 1000–2000 m above sea level are mainly in the Turkish zone, occupying most of its area. The elevation of most of the South Russian and Volga zones does not exceed 1000 m above sea level. The main part of the territories below sea level falls to the east of the South Russian zone. The Balkan and Turkish zones are noted as the most complex relief with a large elevation difference over most of the territory.

The indicators of weather and climatic factors undergo changes depending on the season. The average diurnal temperature range in winter was from 5°C to 11.3°C, in spring from 7.9°C to 15.9°C, in summer from 5.3°C to 19.1°C, and in autumn from 6.5°C to 17.5°C. In each season, the lowest indicators were noted in the Volga and the South Russian zones, and the highest were noted in the Turkish and part of the Israeli zones. The average precipitation rate in winter ranged from 15.6 mm/month to 186 mm/month, in spring from 2.9 to 123.3 mm/month, in summer from 0 to 113.6 mm/month, and in autumn from 3.2 to 149.2 mm/month. In all seasons of the year, the lowest indicators were noted in the east of the South Russian zone and the highest in the west of the Balkan zone. A significant decrease in the precipitation rate to minimum values was noted throughout the Turkish zone in summer. In addition, the seasonal change in the average precipitation rate in the Israeli zone is clearly expressed: the maximum rate was recorded in winter and the minimum was observed in summer.

The average number of wet days varied from 5.8 to 18.6 in winter, from 1.8 to 17 in spring, and from 1.7 to 14.4 in autumn. The annual average was 2.5–14.1. According to annual average data, this indicator underwent spatial changes in accordance with the latitudinal zonality of the Northern Hemisphere and altitudinal zonality, i.e., the number of wet days increased northward and in mountainous regions. The average cloud cover index in winter was 38.6%–86.6%, and in spring, it was 29.8%–72.4%. The least cloud cover in both seasons was observed in the south of the Turkish and Balkan zones and throughout the Israeli zone. When moving from south to north, this indicator increased, which was most pronounced in the Balkan and Turkish zones, while the average cloud cover in the Volga zone was lower than that in the South Russian zone. The average wind speed in the test zones varied from 0.8 to 8.6 m/s in winter and from 1 to 4.9 m/s in summer. At the same time, the lowest average speeds of the prevailing winds in both seasons were typical for the western part of the Balkan and the south of the South Russian zones and the highest for the north-eastern part of the South Russian and Volga zones. The average vapor pressure in the air and the average number of frost days were the limiting factors only in summer. The vapor pressure factor varied from 4.2 to 25.8 hectopascal (hPa) and was inversely related to the elevation above sea level. The highest rates, over 20 hPa, were noted in the Israeli and southwest Turkish zones. In most areas of the remaining zones, the vapor pressure was in the range of 10–15 hPa. The number of frost days was almost equal to zero in the summer, and only in high-mountain areas in the south of the South Russian zone did these indices rise to 13.5 days/month. The average temperature of the wettest quarter of the year ranged from −8.7°C–24.3°C. Indicators above 20°C were typical for most of the Volga zone, the South Russian zone, and the north of the Balkan zone. Values of 0°C–5°C were observed mainly in the mountainous regions of the Balkan and Turkish zones. In the Israeli zone, the indicator of this factor ranged within 10°C–15°C.

The risk of LSD spread prediction based on the developed seasonal and annual models was performed for the Saratovskaya oblast of the Russian Federation. The oblast is located southeast of the East European Plain, in the northern part of the Lower Volga region, between 50 and 53° of northern latitude and 42 and 51° of eastern longitude. The area occupied by the oblast reaches 101.2 thousand square kilometers. It borders the Penzenskaya, Ulyanovskaya, Samarskaya, Orenburgskaya, Volgogradskaya, Voronezhskaya, and Tambovskaya oblasts of the Russian Federation and the West Kazakhstan region (Kazakhstan). The Volga River flows through the oblast, dividing it into two subregions: the Right Bank, located to the west of the river, and the Left Bank, located to the east. The region is divided into 38 municipal districts, 20 of which are located in the Right Bank and 18 in the Left Bank. The capital of the oblast is Saratov.

**Figure-4:**
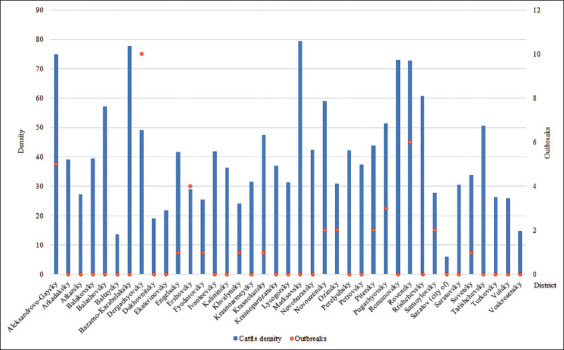
Cattle density, heads per 10 km^2^, (2011–2020) [16] and the number of registered outbreaks during LSD epizootic period (2017–2019) in the districts of the Saratovskaya oblast [19–21].

LSD outbreaks in the region were registered from 2017 to 2019. During these years, there were 41 outbreaks, 29 of them in 2017, one in 2018, and 11 in 2019 (Figure-4) [19–21]. The summer months registered 33 (80.5%) outbreaks, the autumn months showed 8 (19.5%) outbreaks, and there were no cases of the disease in susceptible animals in spring and winter.

Cattle density in the Saratovskaya oblast during the study period ranged from 6 to 79.5 heads/10 km^2^. More than 70 heads/10 km^2^ were noted in two districts of the Right Bank (Bazarno-Karabulaksky and Romanovsky) and in three districts of the Left Bank (Aleksandrovo-Gaisky, Marksovsky, and Rovensky) (Figure-4). The values of road density in the region are in the range of 0–2117 m/km^2^, and the highest density is observed in and near the settlements.

**Figure-5:**
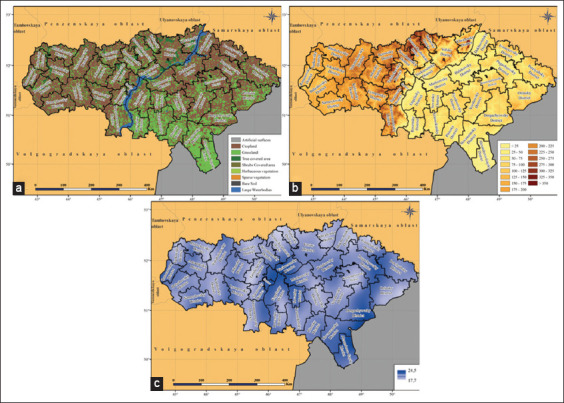
Environmental conditions on the territory of the Saratovskaya oblast: (a) land cover [18], (b) elevation above sea level (in meters) [13], and (c) density of water bodies (m^2^/km^2^) [14] (Source: Base map adopted from NextGIS [22] and GADM [23]).

The land cover is mainly represented by cropland (68.1% of the territory) and grassland (24.2%), with the latter predominantly concentrated in the southeastern part of the oblast. Tree-covered areas occupy 4% of the territory and are mostly in the Right Bank. Snow and glaciers are completely absent, and the share of other types of land cover is extremely small, each of which equals <1% of the territory of the region (Figure-5a). The elevation of the territory of the Saratovskaya oblast above sea level varies from 8 to 368 m. At the same time, the difference between the Right Bank and the Left Banks is clearly expressed. The Right Bank of the oblast is more elevated than the Left Bank. Along the right bank of the Volga, there is the Volga upland with altitudes from 200 m above sea level and higher, up to the maximum value in the oblast. In the direction to the west, it gradually descends to the Oka-Don plain with heights of 100–150 m above sea level. The Left Bank is lower. Most of it is occupied by the Syrt plain with heights of 50–100 m above sea level, bounded by the spurs of the Syrt upland (150 m above sea level) from the east. To the southeast of the oblast, the Syrt Plain passes into the Caspian lowland (<25 m above sea level). A common feature of the Syrt Plain and the Caspian lowland is a decrease in the average elevation to the south and southeast, as well as to the west toward the Volga Valley (Figure-5b) [13]. The average distance from the banks of the water bodies does not exceed 85 m, and the density of the water bodies ranges from 17.7 to 24.5 m^2^/km^2^ (Figure-5c) [14].

The climate prevailing in the territory of the Saratovskaya oblast is temperate continental with hot summers and cold winters. The continentality of the climate increases in the direction from north to southeast. In this direction, the diurnal range of air temperatures increases in each season. In winter, this environmental factor was in the range of 5.2°C–7.1°C, in spring 9.6°C–10.9°C, in summer 11.6°C–13.6°C, and in autumn 7.4°C–9.6°C during the study period. A similar situation is observed for the average precipitation rate: the climate of the Left Bank is more arid, and in each season of the year, the average precipitation rate decreases toward the southeast. The minimum average level of precipitation during the study period was 24.3 mm/month in winter, 22.6 mm/month in spring, 20.2 mm/month in summer, and 26.5 mm/month in autumn. The maximum was 44, 42.3, 49.5, 42.2 mm/month, respectively. In winter, precipitation is mainly represented by snow and in other seasons by rain. In general, the zonality of precipitation distribution is quite clearly expressed on the territory of the region. The spatial distribution of the average number of wet days and the average level of precipitation decreases in the southeasterly direction. In winter, the average number of wet days was 10.8–15.7 days/month; in spring, 7.9–11.3 days/month; and in autumn, 7.8–11.5 days/month. The average annual range was 8.1–11.8 days/month. The cloud cover index in winter was 70.1%–81.3%, and in spring, 55.7%–62.3% decrease in the east and southeast directions. Strong winds are very rare in this oblast. In winter, the average wind speed was 3.6–4.6 m/s, and in summer, it was 2.9–3.4 m/s. In winter, the lowest wind speeds are typical for the northeastern parts of the oblast, and the highest are typical for the northwestern parts. In summer, the weakest winds are observed in the west of the region, and the strongest are on the Left Bank, over the Syrt plain. The saturated vapor pressure was in the range of 12.9–14.2 hPa. The maximum pressure is typical for the Oka-Don Lowland in the western part of the oblast and over the Volga valley, and the minimum pressure is typical for the southeastern part of the region. There are no frost days during summer. The average temperature of the wettest quarter of the year in the Right Bank was within 15°C–20°C, in the Left Bank 20°C–23.1°C, which corresponds to the maximum in the oblast.

All of the seasonal MaxEnt models built on the basis of these environmental factors showed good average test AUC results. They were 0.961 ± 0.027 for the winter model, 0.922 ± 0.025 for the spring model, 0.802 ± 0.023 for the summer model, and 0.906 ± 0.032 for the autumn model. In the case of the annual model, the average AUC was lower, 0.781 ± 0.014, but the predictive ability remained high.

The results of assessing the relative gain of various environmental factors to the MaxEnt models built for all test areas using the heuristic analysis method and its refinement using the permutation test are shown in Table-3.

**Table-3:** The relative gain of environmental factors (independent variables) in MaxEnt models for all test zones, calculated by heuristic analysis and reevaluated by permutation test. The results are presented as the resulting drop in training AUC, normalized to percentages.

Variable	Season models				Annual model

	Winter model	Spring model	Summer model	Autumn model	
Average distance to nearest water bodies (of all types)	1	1.2	3.2	2	5
Cattle density	**10.5**	2.2	**8.7**	7.5	**15.6**
Cloud cover	5.2	6.2			
Density of roads (of all types) network	**13.6**	2.1	3.3	6.9	6
Density of water bodies (of all types) network	6.3	**18.4**	**36.3**	6	**33.5**
Diurnal 2 m temperature range	5.4	9.4	2.2	**18.1**	
Dominant type of land cover	3.7	2.5	3	0.8	11.3
Elevation above sea level		3.1			
Frost days			2.6		
Mean temperature of wettest quarter (bio_8)					**14.6**
Mean wind speed	9.8		3.8		
Precipitation rate	**42.4**	**23.8**	**33.3**	**40.1**	
Vapour pressure			3.7		
Wet days	2	**31.2**		**18.7**	14

Factors whose gains exceed the average value of gains of all factors in the corresponding model are highlighted in bold. Variables that retain their gain exceed the average gains of all factors in the corresponding model in three or more models are highlighted in gray.

The set of variables in the MaxEnt models for the test zones changes with the seasons. Three predictors with a gain that exceeds the average gain of all other factors of the model can be distinguished in each model after the permutation test. During winter, the highest gains in the LSD risk spread model were cattle density, road density, and especially the average monthly precipitation rate. The gain of other variables in this model of pathogen spread had low significance. During the spring, the highest gains were the average number of wet days per month, the average precipitation rate per month, and the density of the water bodies. In summer, the highest gains were the density of water bodies, the average monthly precipitation rate, and, to a much lesser extent, cattle density. In autumn, the highest gains in the model had the following weather factors: the average precipitation rate per month, the average monthly number of wet days, and the average diurnal temperature range, the latter two with almost similar values. In the annual LSD spread risk model, the most significant gain was made by the density of water bodies, the cattle density, and the average temperature of the wettest quarter.

**Figure-6:**
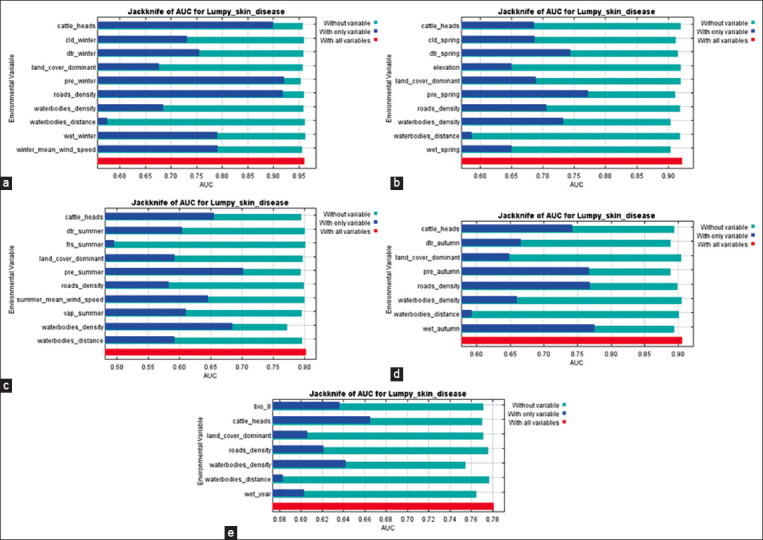
Influence of environmental factors (variables) on the overall quality of the constructed LSD risk spread models. (a) winter model, (b) spring model, (c) summer model, (d) autumn model, and (e) annual model.

The significance of environmental variables for the quality of the constructed models, calculated using the jackknife method, is shown in Figure-6 (only graphs with the results of calculating AUC on test data are shown).

According to jackknife analysis, in the winter, spring, and summer models of the LSD spread risk, the most significant variables were the average precipitation rate per month and road density in autumn. With the omission of variables from the models, the most significant decrease in the quality of modeling was observed: in the winter model, except for the average precipitation rate per month; in the spring and summer models, except for the density of water bodies; and in the autumn model, except for the diurnal temperature range. In the annual model, the most significant variable was the cattle density; however, the greatest decrease in model quality occured when the density of water bodies was excluded. These data correspond well to the heuristic analysis data (Table-3).

**Figure-7:**
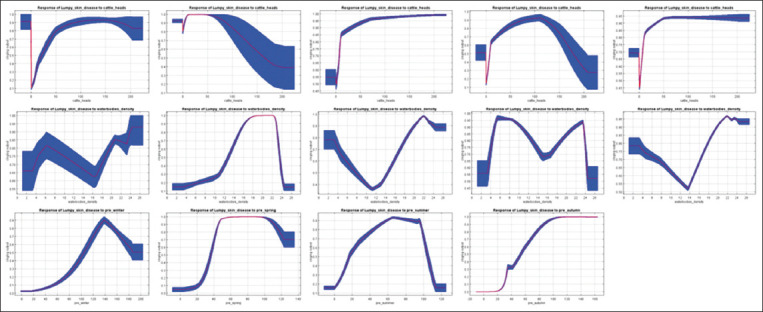
Response curves of variables showing above-average gain in three or more MaxEnt models. The top row is cattle density, the middle row is the density of the water bodies, and the bottom row is the average precipitation rate per month. Columns, from left to right: winter, spring, summer, autumn, and annual models. The X-axis shows the indicators of variables, and the Y-axis shows the index of the spatial distribution of LSD pathogen. Blue shows the standard deviation.

The influence pattern of the environmental factors used in the models was displayed by the response curves of the corresponding variables. Each curve demonstrates how the spatial distribution index of the pathogen changes with a change in the specific variable, provided that the values of the other variables remain at the level of their average sample value. Figure-7 shows the response curves of variables with an above-average gain in three or more MaxEnt models, i.e., environmental factors that significantly influence the LSD spread risk throughout most of the year. It shows how the index of the pathogen’s spatial distribution varies with the change of seasons, especially in the case of the change in the water body density factor. If, in spring, the graph of this index demonstrates the presence of an increased risk at values of ≈ 13–23.5 m^2^/km^2^, then in other seasons, it has a complex geometry with pronounced pessimum and optimum. At the same time, in winter and autumn, the risk was increased at any density of water bodies, whereas in summer, the index of the spatial distribution of the pathogen in the range of ≈ 8–14.5 m^2^/km^2^ was below the average value. The index curve of the water body density in the annual model, although it has a pronounced pessimum with a water body density index of approximately 13.5 m^2^/km^2^, always remains above 0.5. The change in the other variables was not so radical. The risk of the LSD outbreak increased in winter and autumn with cattle densities above 10 and 20 heads/10 km^2^, respectively, and in spring and summer, it increased regardless of density. This pattern is also confirmed by the index curve in the annual model. The curve of the spatial distribution index that depends on the average monthly precipitation rate is above 0.5 in winter in the range of ≈ 105–185 mm/month, in spring at values above ≈ 35 mm/month, in summer in the range of ≈ 15–105 mm/month, and in autumn at values above 50 mm/month.

**Figure-8:**
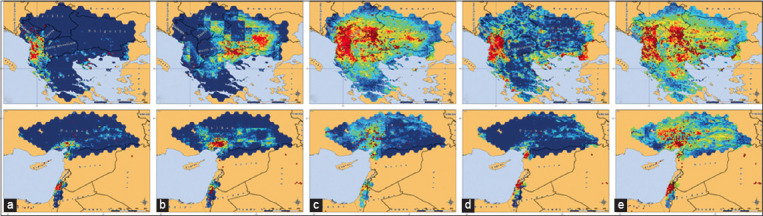
Presence probability distribution of the LSD pathogen in the Balkan zone (upper row), and Turkish and Israeli zones (lower row), visualized by the MaxEnt model. (a) Winter model, (b) Spring model, (c) Summer model, (d) Autumn model, (e) Annual model. Dark blue indicates extremely low probability, light blue indicates below average probability, green indicates average probability, yellow indicates above average probability, and bright red indicates high probability. Burgundy dots are registered outbreaks of LSD in cattle (Source of base maps: NextGIS [22] and GADM [23]).

**Figure-9:**
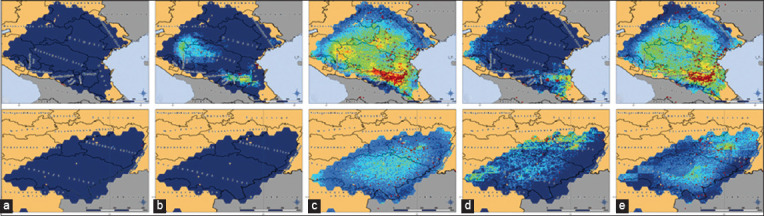
Presence probability distribution distribution of the LSD pathogen in the South Russian zone (upper row) and the Volga zone (lower row), visualized by the MaxEnt model. (a) Winter model, (b) Spring model, (c) Summer model, (d) Autumn model, and (e) Annual model. Dark blue indicates extremely low probability, light blue indicates below average probability, green indicates average probability, yellow indicates above average probability, and bright red indicates high probability. Burgundy dots are registered outbreaks of LSD in cattle(Source of base maps: NextGIS [22] and GADM [23]).

On the basis of the calculated gain of the selected factors and the character of their influence, it is possible to visualize the spatial distribution of the LSD pathogen and the occurrence of an LSD outbreak for different seasons and annually (Figures-8 and 9). Thus, among the test zones, the highest risk of a disease outbreak in winter was noted in the west of the Balkan zone, in the southwest of the Turkish zone, and in the north of the Israeli zone. At the same time, the medium-risk zone occupied a small area around high-risk territories; in general, the risk of LSD spread was low across all test areas. In spring, there was an expansion of high- and moderate-risk areas in the Balkan and Turkish zones, while the greatest risk in the Balkan zone shifted to the east into the territory of North Macedonia, Bulgaria, and Greece. Also, the emergence of moderate-high risk areas in the part of the South Russian zone was discovered (Krasnodarsky and Stavropolsky krays, the Republics of Ingushetia and Chechnya). The risk of LSD outbreaks in the Volga zone in winter and spring remained low. In summer, high- and medium-risk areas expanded significantly and reached their largest size throughout the year. A high risk of LSD outbreaks was observed in almost all territories of the Balkan zone during this season. High-risk areas appeared for the 1^st^ time in a year in the South Russian zone, and most of the Volga zone became an area of medium risk for LSD spread. In autumn, the risks began to decrease again. In the Balkan zone, high-risk areas were concentrated in the western part, whereas small high-risk areas remained at the edge of the southeastern part. The highest risk was again noted in the southwestern part of the Turkish zone. In the South Russian zone, the area with a high risk of LSD spread was shifting to the territory of the Dagestan Republic. In the Volga zone, territories along its eastern border became unsuitable for the spread of the pathogen, and a small area of increased risk of LSD spread was formed in its north. The annual model of the probability of virus presence repeated the distribution of the probability calculated in the seasonal models.

**Figure-10:**
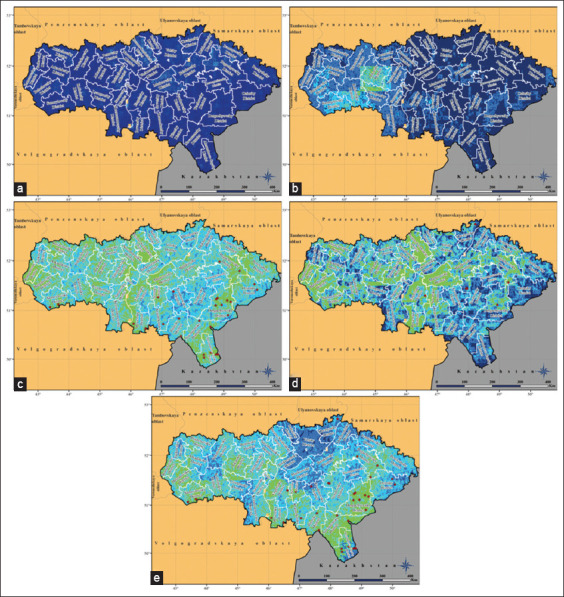
Presence probability distribution of the LSD pathogen in the Saratovskaya oblast, visualized by the MaxEnt model. (a) Winter model, (b) Spring model, (c) Summer model, (d) Autumn model, (e) Annual model. Dark blue indicates extremely low probability, light blue indicates below average probability, green indicates average probability, yellow indicates above average probability, and bright red indicates high probability. Burgundy dots are registered outbreaks of LSD in cattle (Source: Base map adopted from NextGIS [22] and GADM [23]).

The extrapolation of the seasonal and annual models to the territory of the Saratovskaya oblast provided a more detailed picture of the LSD spread risk in this region (Figure-10). According to the data obtained, the spread risk of this disease was at an average level compared with the highest-risk areas located in the Balkan or South Russian zones. Simultaneously, the distribution of risk in time and, in most cases, in space was not uniform, which makes it possible to identify areas with the greatest risk of LSD outbreaks in different seasons. In winter, the risk value was at an extremely low level throughout the oblast, and there were only minor fluctuations, as shown in Figure-10a. In spring, a low risk of outbreaks persisted in most of the oblast, especially in the Left Bank and the northwestern part of the Right Bank. It was slightly higher in the central and southern parts of the Right Bank (Figure-10b). Summer on the territory of the Saratovskaya oblast appeared to be the most favorable for the spread of the LSD pathogen (Figure-10c). The zones of greatest risk in the oblast from June to August inclusive were concentrated in its west (Romanovsky and Balashovsky districts), in the center (Bazarno-Karabulaksky, Tatishevsky, Marksovsky, Engelssky and Rovensky districts), and east (Ivanteevsky, Perelyubsky, Pugachyovsky, Dergachyovsky, Novouzensky, and Aleksandrovo-Gaisky districts). In autumn, the pattern of spatial distribution of the LSD spread acquired the highest heterogeneity of the year (Figure-10d). The risk on the border with Kazakhstan was significantly reduced, and the areas with the highest risk in the oblast shifted to its central geographical axis, especially to the Bazarno-Karabulaksky district on the right bank and to three districts located on the left bank of the Volga valley: Marksovsky, Engelssky, and Rovensky. In addition, the risk remained average in the western districts. Visualization of the annual risk model demonstrates the location of the largest areas with the highest risk for the oblast in its southeastern part on the territory of the Dergachyovsky, Novouzensky, and Aleksandrovo-Gaisky districts. In contrast, the area with the lowest risk (low across all test areas) was located in the north of the region (Figure-10e).

## Discussion

The complexity of modeling the spatiotemporal distribution of transmissible infectious disease pathogens and predicting the quantitative probability of their spread risk is closely related to the distribution of susceptible organisms and all possible vectors. In the case of LSD, the distribution of its pathogen is also influenced by human economic activities, such as the movement of infected cattle and virus carriers, vaccination campaigns, and vector eradication. These circumstances indicate the presence of many factors that can influence the spread of pathogens.

In this study, we examined the influence of various groups of environmental factors on the seasonality of pathogen spread in LSD-endemic areas. Affected areas selected for training models are located in different climatic zones and have different topography and different types of dominant land cover. The cattle density varies significantly. To improve the quality of modeling, we used weather data for the same period (2011–2020) when LSD outbreaks were registered. We also used the number of cattle in the municipal districts of Saratovskaya oblast data related to the same period to build a predictive model.

In general, the data of this study are consistent with the results of other authors obtained by analyzing the spread of the LSD pathogen despite differences in research approaches. Thus, in the works of Alkhamis and VanderWaal [35] and Allepuz *et al*. [36], devoted to LSD spread in the Eastern Mediterranean, it was shown that the average annual precipitation rate, temperature indicators, and cattle density are among the most significant risk factors. A connection between the average precipitation rate and LSD outbreaks was found in Ethiopia [37]. A significant influence of the change of seasons, along with some other factors, on LSD virus seroprevalence was established on the basis of statistical data related to Egypt [38, 39]. In Turkey during the 2014–2015 LSD epizootic in locations of outbreaks, positive samples were obtained from mosquitoes whose life cycle is associated with open water bodies [40]. In 2021–2022, LSD cases were reported in Thailand. According to the authors’ assumption, the disease spread rate in the absence of movements of susceptible animals indicates the participation of insect vectors in the epizootic process [41]. From studies of the LSD epizootic in areas with a temperate climate, it should be noted that the virus genetic material was isolated from *Arthropoda* in Kazakhstan [42] and some regions of Russia [8, 43]. During the epizootic analysis of LSD outbreaks in 2021 in Mongolia, it was found that the average direction of their distribution closely corresponds not only to the general direction of transport routes but also to the average direction of the winds prevailing in the area, which confirms the fact of LSD pathogen transmission by airflows spreading arthropods over long distances [7].

Thus, arthropod vectors can play a significant role in the rooting of LSD and the development of stationary morbidity in the area if conditions are favorable for them, even if such conditions persist not all over the year. Conducting anti-epizootic measures to prevent the spread of this disease, including vaccination campaigns, reduces the risk of outbreaks but does not completely exclude it because the possibility of re-introduction of the pathogen remains. This determines the importance of continuous monitoring of environmental conditions in areas at risk of LSD spread.

## Conclusion

According to the modeling results, the risk of LSD spread during different seasons is determined by a total of fourteen environmental factors. In every model of the LSD spatial distribution, several predictors with gain exceed the average gain of all variables in this model. It is also possible to identify variables that retain high gains in several models, i.e., throughout several seasons. These include cattle density (highest gain in the winter and summer models), the density of water bodies (spring and summer models), and the average monthly precipitation rate (all seasonal models).

The high gain of the first two predictors in seasonal models is confirmed by their results in a separately calculated annual model. The mean precipitation factor, which did not pass the preliminary test at generation of the annual model, was displaced by the average wettest quarter temperature predictor (BIO8), which was not used in the seasonal models. The influence of the wet days per month factor was slightly less pronounced. This variable passed the multicollinearity test in three seasonal models, and in the spring and autumn models, its influence is among the most significant. In the annual model, its influence is at the average level.

The effect of temperature variables on LSD spread risk is heterogeneous. Preliminary analysis was passed only by the average diurnal temperature range factor. This factor is in all seasonal models but exceeds the average value only in the autumn model and approaches it in the spring model. In summer, it is the least significant of the factors that passed a preliminary statistical analysis. In the annual model, it is absent, displaced by the above-mentioned indicator BIO8 and the precipitation rate factor.

Visualization of the LSD pathogen presence probability in the considered test zones, calculated using the mentioned predictors and retrospective data on disease outbreaks, shows that areas with a risk of LSD spread change in size with the seasons. In summer, they reach the largest areas, whereas in winter, the danger remains only in areas with a warm Mediterranean climate.

Compared with areas with the highest risk, the Saratovskaya oblast has a relatively low risk of LSD spread. The summer period is the most favorable for pathogen spread. The probability of the virus’s presence is distributed practically uniformly throughout the oblast in this season. The central and western parts of the oblast are more at risk in autumn. Spring is unfavorable for epizootic events. In winter, the risk of LSD outbreaks is practically reduced to zero in relation to the factors considered.

## Authors’ Contributions

DP, VA, and LP: Designed the study, performed the modeling, and wrote the manuscript. DP and OP: Collected and analyzed the data and reviewed the manuscript. OC and KM: Performed statistical analysis, compiled the figures and graphs, and reviewed the manuscript. NS: Analyzed the data and drafted and reviewed the manuscript. All authors have read, reviewed, and approved the final version of the manuscript.
